# Supplementary material for: Neural network models for sequence-based TCR and HLA association prediction
Source: PLoS Comput Biol. 2023 Nov 20;19(11):e1011664. doi: 10.1371/journal.pcbi.1011664 (PMC10695368; doi:10.1371/journal.pcbi.1011664)
Supplement: Attachment — Submitted filename: PLOS_CB_response.pdf [file pcbi.1011664.s002.pdf]

Response on Neural Network Models for Sequence-based TCR and HLA Association Prediction

October 3, 2023

We apologize that in previous submission, some content explaining certain details of the model training and the computation of between-individual HLA distances was left out. We have added the content back, part to the Materials and methods section in the main text, and part to Sections 3 and 4 in S1 Appendix.

Reviewer #1

Liu et al. proposed to use neural network models to predict the association between T cell receptors (TCR) and human leukocyte antigens (HLA) with their amino acid sequences. After constructing the training data of positive and negative pairs, they selected the tuning parameters in the deep learning model to predict associations. Benchmarking shows better performance than the existing method CLAIRE. The proposed DePTH differs from CLAIRE by different training data, and DePTH allows unseen HLA alleles in training data since it directly inputs the sequences. In the application of immune checkpoint blockade, the authors showed that DePTH-predicted TCR-HLA associations could be useful as biomarkers associated with clinical outcomes. The paper is well-written, the analyses are comprehensive, and the scientific contribution is strong. I list some minor comments below that may further improve the manuscript.

"Table C: Performance of DePTH on leave-one-out experiments" The AUC is above 0.64, specificity is above 0.92, but recall/sensitivity is below 0.31. This suggests a better cutoff other than 0.5 may be selected to balance specificity and sensitivity in leave-one-out experiments. This may help predict new TCRs and HLAs unseen during training.

Thanks a lot for the suggestion. We agree that optimal prediction score cutoffs for the leave-one-out experiments do appear to be different from the default value of 0.5. We have mentioned this in the revised manuscript (page 7) and listed the values of recall and

specificity on test data under different prediction score cutoffs for the three leave-one-out experiments in Table 1 below. This table is also added to S1 Appendix as Table D.

Table 1: Performances of DePTH models trained separately for three leave-one-out experiments based on Emerson data. This table lists the recall and specificity at different prediction score cutoffs.

	HLA-B*08:01		HLA-B*07:02		HLA-C*07:01	
cutoff	recall	specificity	recall	specificity	recall	specificity
0.1	0.88	0.21	0.88	0.30	0.84	0.34
0.2	0.73	0.43	0.68	0.55	0.68	0.59
0.3	0.55	0.64	0.50	0.75	0.51	0.76
0.4	0.40	0.81	0.35	0.89	0.40	0.86
0.5	0.26	0.92	0.22	0.95	0.31	0.92
0.6	0.16	0.97	0.16	0.98	0.25	0.95
0.7	0.10	0.99	0.08	0.99	0.18	0.97
0.8	0.05	1	0.05	1	0.12	0.99
0.9	0.02	1	0.02	1	0.06	1

"The AUC to distinguish these 54 TCR-HLA pairs from the negative pairs from Emerson test data is 0.87". The proposed DePTH with Emerson data performs well for the solved TCR-pMHC-I structures. I wonder how CLAIRE or DePTH with McPAS data performs. This can be a potential comparison as well.

Thanks a lot for the suggestion. Since models trained on McPAS data do not generalize to the Emerson data. We applied DePTH trained with McPAS data (DePTH-McPAS) and CLAIRE (which was trained with McPAS data) to distinguish 54 positive TCR-HLA pairs (from the solved TCR-pMHC-I structures reported by Szeto et al.) versus negative pairs from McPAS test data.

The AUCs for DePTH-McPAS and CLAIRE are 0.68 and 0.65, respectively. In the figure below (also added to S1 Appendix as Fig. E), subfigure (A) shows the violin plots of prediction scores from the two models on the positive/negative pairs of McPAS test data, and the 54 external positive TCR-HLA pairs from Szeto et al..

Since HLA-A*02:01 is involved in more than 55% of the TCR-HLA pairs from the Szeto pairs or the McPAS negative test pairs, we also evaluated the results for the TCR-HLA pairs involving HLA-A*02:01 versus other TCR-HLA pairs. For the group involving HLA-A*02:01, DePTH-McPAS and CLARE achieved an AUC of 0.79 and 0.97, respectively. For the other TCR-HLA pairs, the AUC by DePTH-McPAS and CLARE are 0.60 and 0.30, respectively. Subfigure (B) in the figure below shows these results in a barplot. In summary, DePTH-McPAS has lower performance than CLAIRE on the TCR-HLA pairs involving HLA-A*02:01, the most frequent HLA-I allele in McPAS training data, but has

better performance than CLAIRE for other TCR-HLA pairs.

We included a brief description of this comparison at the end of the Subsection "DePTH outperforms CLAIRE on most HLAs when trained on the same data" in page 9 of the main text and added more detailed description as S1 Appendix Section 7.

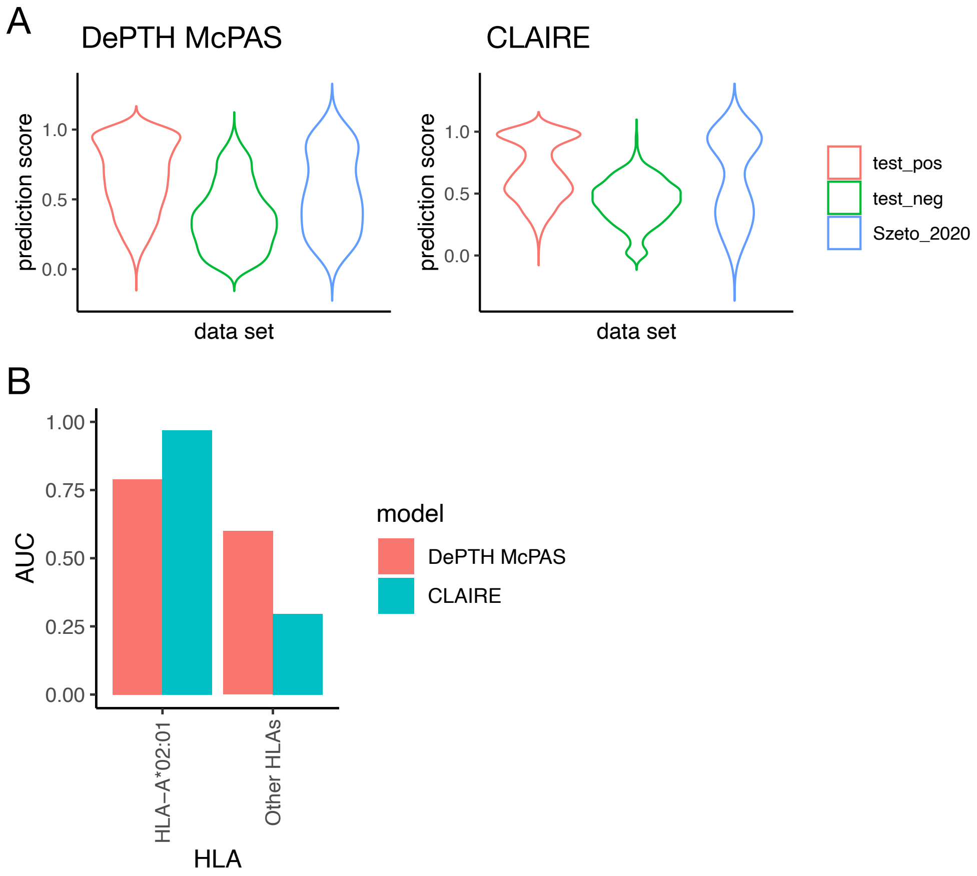

Figure 1: The performances of DePTH trained on McPAS data and CLAIRE on distinguishing Szeto pairs from McPAS negative test pairs. (A) Violin plots for the TCR-HLA association scores predicted by the two models on three sets of TCR-HLA pairs: the positive pairs of McPAS test data, the negative pairs of McPAS test data, and the 54 external positive TCR-HLA pairs from solved TCR-pMHC-I structures provided by Szeto et al. [1]. In the subfigure on the left, the scores are given by DePTH model trained on McPAS training data. In the subfigure on the right, the scores are given by CLAIRE model. (B) Comparison of AUCs when the TCR-HLA pairs in Szeto pairs and McPAS negative test pairs are separated into two groups, one group involving HLA-A*02:01 and the other group involving HLAs alleles other than HLA-A*02:01.

"The validation AUC became stable after ensemble size increased to around 20 (S1 Appendix Fig B)." Based on Fig B, shuffles # 1 and 3 have stable AUCs around 20 models,

but the other two shuffles have AUCs increasing even after 20 models, which suggests more models can be used in the ensemble if computation is not a concern.

Thank you for your suggestion. Yes, the four shuffles showed different patterns. In the revised manuscript, we have quantified the consistency of two ensemble models using an average of 100 replicates to obtain more robust results.

Specifically, we have evaluated the consistency between two ensemble models that have the same ensemble size by the Spearman's correlation of their prediction scores. For ensemble size n, each time we sample two distinct sets of models with n models in each set. Each set of models form an ensemble model, and we compute the Spearman's correlation between the prediction scores given by these two ensemble models on the validation data. Under each ensemble size n, we repeat this procedure 100 times and obtain the average of 100 resulting Spearman's correlation values. We vary the ensemble size n from 1 to 50 and plot the average value of Spearman's correlation v.s. the ensemble size n. As shown in the figure below (also added to the manuscript as S1 Appendix Fig. B(B)), the average correlation reaches 0.976 at ensemble size 20 and only increases moderately after that.

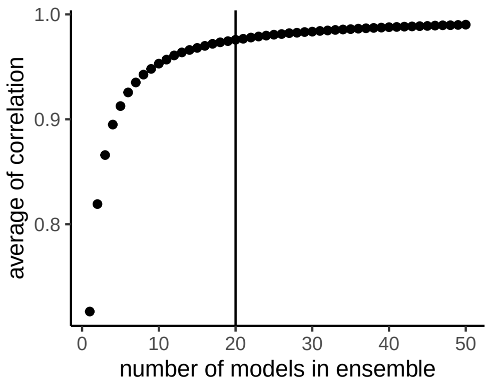

Figure 2: The average of Spearman's correlations of prediction scores on Emerson validation data given by two ensembles of models v.s. the ensemble size. The models in all ensembles are sampled from 100 single models trained on Emerson training data using 100 different sets of random seeds. At each ensemble size n from 1 to 50, each time, two ensembles of n models are sampled from the 100 single models, and Spearman's correlation is computed from the prediction scores given by these two ensembles on Emerson validation data, and this procedure is repeated 100 times. The value on y-axis corresponds to the average of the 100 resulting values of Spearman's correlation.

In terms of computational time, when running on a computing node with Intel Gold 6154 CPU and around 40GB requested memory on computing server, it takes about 4 minutes to finish training one single model. Further increasing the number of models in ensemble will lead to longer total training time. Another potential issue is the size of the package if we have many models. Currently when 20 trained models for HLA-I and 20 trained model for

HLA-II are carried with the package, the package takes about 41Mb. Carrying more models in the package will further increase the package size.

We have added these considerations about choosing ensemble size as S1 Appendix Section 4.3.

Reviewer #2

This manuscript developed a neural network method named DePTH to predict TCR-HLA associations based on their amino acid sequences. The method outperforms the existing method for the similar task for less common HLA alleles. The manuscript shows that DePTH can be used to quantify the functional similarities of HLA alleles. These similarities are shown to be associated with the survival outcomes of cancer patients who received immune checkpoint blockade treatment.

Major:

1. The comparison with random forest is better combined with Section "DePTH models achieve AUC around 0.8 on test TCR-HLA pairs" to give readers a reference on how good an AUC around 0.8 is.

Thanks a lot for the suggestions. We have separated the comparison with random forest into two parts and merged them separately into the subsections "DePTH models achieve AUC around 0.8 on test TCR-HLA pairs" (last paragraph of page 6) and "DePTH makes accurate prediction on TCR-HLA pairs from solved TCR-pMHC-I structures" (second paragraph at page 8), to make the comparison more straightforward for readers to see.

2. (a) Please explain this sentence more clearly: Line 223: "CLAIRE treats HLA as a categorical variable, which limits its ability to generalize to unseen HLAs". For example, how does CLAIRE treat HLA as a categorical variable. (b) Based on this statement, CLAIRE would have very low predictive power when being used to predict the association in a different dataset. But the results seem to show that the performance of DePTH is similar to CLAIRE. What is the explanation?

(a) Thanks a lot for pointing out this confusing statement. Indeed it is not correct to state that "CLAIRE treats HLA as a categorical variable". CLAIRE takes the alphanumeric name of an HLA allele as input to the model (e.g., HLA-A*02:01). It splits the name string into characters and map each unique character to a unique integer. After this process, the HLA name string is converted into a vector of integers, which is passed as input to the next step of the model. We have added these clarifications in the revised manuscript, in the paragraph on the top of page 9.

(b) Since CLAIRE uses the alphanumeric name of an HLA allele as input to its neural

network, it can be applied to HLA alleles not seen in the training data. Its generalization ability will rely on how the similarity in HLA name strings capture the similarity in their associations with TCRs.

We are not sure whether you also refer to the statement in the paper that CLAIRE, which was trained by McPAS data, has low predictive power in Emerson data. This observation was made in the paper of CLAIRE. In our paper, when we did the comparison between DePTH and CLAIRE, we trained DePTH using the same McPAS data that CLAIRE was trained on, and evaluated the performances on set-aside McPAS test data. In this comparison, the performances of DePTH-McPAS and CLAIRE were similar.

3. In section "DePTH outperforms CLAIRE on most HLAs when trained on the same data", please provide the frequencies for all 10 HLA-I alleles.

Thanks a lot for the suggestion. Table 2 below lists the frequency of each HLA of the 10 HLA-I alleles among the pairs in CLAIRE training data that involve HLA-I alleles, and the same table is also added into S1 Appendix as Table E.

Table 2: The frequency for each of the 10 HLA alleles among the TCR-HLA pairs in CLAIRE training data that involve HLA-I alleles.

hla_allele	frequency
HLA-A*01:01	0.042
HLA-A*02:01	0.440
HLA-A*11:01	0.013
HLA-A*24:02	0.024
HLA-B*07:02	0.058
HLA-B*08:01	0.036
HLA-B*27:05	0.012
HLA-B*35:01	0.004
HLA-B*57:01	0.012
HLA-C*07:02	0.081

4. The section on HLA similarity metrics is not easy to follow. It would help if the rationale of this analyses is explained more clearly at the beginning of the section. In particular, what are the clinical implications of HLA similarities (both between individual and individual-level heterogeneity)? How does TCR-HLA pair relate to HLA similarities? Also when reporting the results, please explain how to interpret the results of this analysis and the clinical implication.

Sorry that we did not explain the rationale clearly. The rationale is as follows. Many clinical outcomes may be related to T cell response, such as cancer patients' survival time after immunotherapy. If we expect that HLAs affect clinical outcomes through T cell response,

then two HLAs that are associated with similar sets of TCRs should have similar clinical outcomes. That is why we quantify the similarity of two HLA alleles by their associated TCRs. If they are associated with similar sets of TCRs, they may induce similar T cell response. We have added this explanation at page 5 of revised manuscript.

It becomes a little bit more complicated when we consider the HLAs of each person because each person has multiple HLA alleles.

In terms of between individual HLA similarity, the rationale is that if two persons have similar sets of HLA alleles, they may interact with similar TCRs, which may lead to similar immune response to tumor and thus similar survival outcomes.

For individual-level heterogeneity, the rationale is that if a person has more diverse HLA alleles, we expect that her/his HLA proteins can interact with more TCRs, and thus may lead to better immune surveillance to tumor. Therefore, more diverse HLA alleles may lead to better survival outcome.

We have revised the subsection "HLA similarity metrics defined based on DePTH prediction are associated with survival outcome of cancer patients treated with Immune Checkpoint Blockade (ICB)" (page 10 and page 11) to interpret the results and the clinical implication.

5. What are the covariates in Fig 4? Please provide a more complete description of the survival analyses.

Thanks a lot for pointing this out. We have added the covariates information in the caption for Fig. 4 (page 11). The covariates include age, log-transformed mutation burden, and drug type (CTLA-4, PD-1, or both).

6. Some parts of the results section involve a long description of the analysis procedure. It may be helpful to accentuate the main message by putting some description of the procedure in the method section and providing more rationales.

Thanks a lot for the suggestion. We have moved the details on computing the HLA metrics into the Materials and methods section and the S1 Appendix Section 9, and also moved some description on data processing steps to S1 Appendix Section 8. To further accentuate the main message, in Subsection "HLA similarity metrics defined based on DePTH prediction are associated with survival outcome of cancer patients treated with Immune Checkpoint Blockade (ICB)", we added more explanation on the rationale in the beginning and also rewrote a large proportion of other content.

Reviewer #3

The authors propose a method called DePTH for predicting TCR-HLA paring that uses the sequence information of HLA alleles (contrasting the competing method using HLA as a categorical variable), thus allow the prediction to generalize to rare and unseen HLAs.

Using the Emerson data to train the model with selective positive associations (co-occurrences of TCR and HLA) and sampled negative pairs as controls. Specifically, they selected 6,423 associated TCR-HLA pairs out of 742,832,595 TCR-HLA pairs involving HLA-I alleles and 11,037 associated TCR-HLA pairs out of 1,136,096,910 TCR-HLA pairs involving HLA-II alleles. They show DepTH outperformed the competing methods in prediction accuracy and further validated using an independent dataset by Szeto et al. The method has the potential to study rare HLAs. They show the method has moderate prediction accuracy for unseen pairs.

The study could be improved if examples of clinical utility can be demonstrated more convincingly. For example, in Chowell dataset, is there any novel findings of predicted TCR-HLA pairs associated with patient survival beyond what was reported in the original paper?

Thank you for your suggestion. The Chowell et al. 2018 study reported the HLA heterozygosity status (0 or 1) is associated with survival time and we have found similar conclusion (Fig 3(C), Fig 4 in main text). One novel aspect of our method is that it allows us to define similarity across individuals and then perform clustering and kernel based regression (Fig 3(A-B), Fig 4 in main text).

In addition, in the revision, we also evaluated associations between HLA similarity/heterozygosity and survival outcomes in The Cancer Genome Atlas (TCGA) cohort and found our method can identify significant associations between HLA metrics and survival outcomes that were missed when only considering HLA heterozygosity.

On TCGA data, we focused on two kinds of survival outcome endpoints, overall survival (OS) and progression-free interval (PFI), which are suggested by Liu et al. 2018[2] as being derived relatively accurately using the available data. We ran analysis for each cancer type separately. For association tests using MiRKAT-S, we consider between-individual HLA-I distances "dist_AA", "dist_DePTH" and "dist_DePTH_breadth". For association tests using Cox regression, we consider individual-level HLA-I heterozygosity metrics including homozygous (0/1), "mean_AA", "mean_DePTH_cor", "mean_DePTH_set" and "DePTH_breadth". For each kind of survival endpoint, we only consider the cancer types for which the corresponding survival endpoint is recommended to use, according to Table 3 of [2]. In terms of covariates, we try two cases, one with no covariate and the other one with all covariates, including age, pathological stage (for certain cancer types, clinical stage is used instead) and log transformed tumor mutation burden. More details are added as S1 Appendix Section 12.

The tables below list the resulting p-values from each of (MiRKAT-S, Cox regression)*(OS,

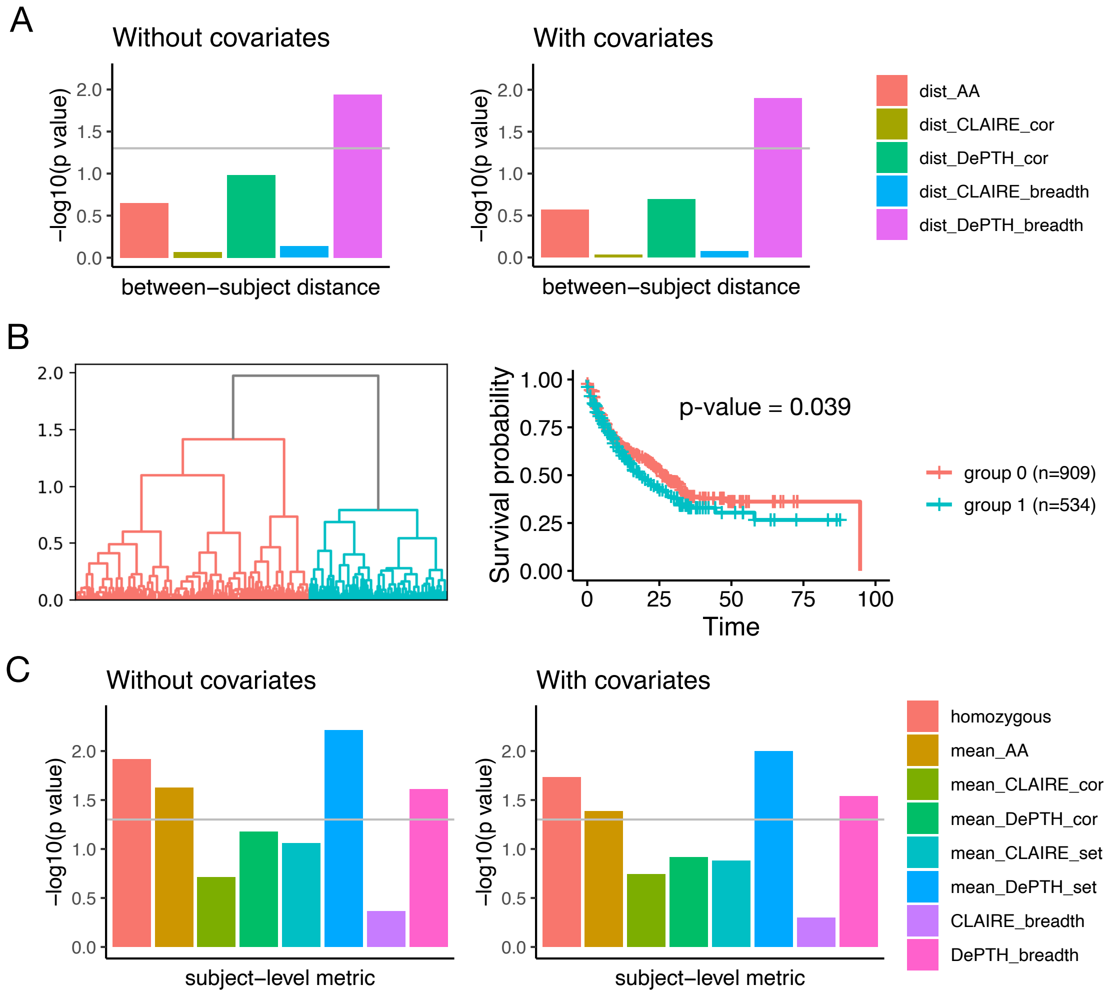

Figure 3: **Association between HLA-based metrics and survival outcomes of 1,443 cancer patients.** (A) and (B) summarize the results using between-individual HLA-I distance metrics. (A)-log10(p-value) of kernel regression using five different distance matrices with or without covariates. The covariates include age, log-transformed mutation burden, and drug type (CTLA-4, PD-1, or both). The grey horizontal line corresponds to p-value 0.05. (B) The dendrogram of hierarchical clustering where the distance is defined by "dist_DePTH_breadth", and the survival curves of the two subgroups identified by cutting the dendrogram tree. The p-value is computed from Cox proportional hazard regression. (C) -log10(p-value) from Cox regression for the associations between individual-level HLA-I heterozygosity metrics and survival outcome, with or without covariates. The covariates include age, log-transformed mutation burden, and drug type (CTLA-4, PD-1, or both). The grey horizontal line corresponds to p-value 0.05.

PFI) combinations, and only the situations with p-values below 0.05 are listed.

The situations with p-value < 0.05 from running kernel regression MiRKAT-S on TCGA data are listed in Tables 3& 4. We see that "dist_AA" and our metrics capture significant associations for different cancer types.

Table 3: Situations with p-value < 0.05 from running MiRKAT-S on TCGA data, with OS as survival endpoint.

covariates	cancer type	HLA-I distance	pvalue
no covariate	KIRC	dist_DePTH_cor	0.0438
no covariate	KIRC	dist_DePTH_breadth	0.0129
no covariate	BLCA	dist_DePTH_cor	0.0328
no covariate	BLCA	dist_DePTH_breadth	0.0259
no covariate	STAD	dist_AA	0.0149
age+stage+log1p(mut)	BLCA	dist_DePTH_cor	0.0232
age+stage+log1p(mut)	BLCA	dist_DePTH_breadth	0.0072
age+stage+log1p(mut)	STAD	dist_AA	0.0133

Table 4: Situations with p-value < 0.05 from running MiRKAT-S on TCGA data, with PFI as survival endpoint.

covariates	cancer type	HLA-I distance	pvalue
no covariate	STAD	dist_AA	0.0222
no covariate	LIHC	dist_AA	0.0254

The situations with p-value < 0.05 from running Cox regression on TCGA data are listed in Tables 5& 6. The HLA heterozygosity status used by Chowell et al. fails to any identify associations. In contrast, "DePTH_breadth" identify associations with OS in BLCA and LUAD, and several other metrics identify associations with PFS in LUAD and LUSC.

Table 5: Situations with p-value < 0.05 from running Cox regression on TCGA data, with OS as survival endpoint.

covariates	cancer type	HLA-I metric	pvalue
no covariate	BLCA	DePTH_breadth	0.0177
age+stage+log1p(mut)	LUAD	DePTH_breadth	0.0223
age+stage+log1p(mut)	BLCA	DePTH_breadth	0.0110

For the Chowell 2018 cohort, all patients received immune checkpoint blockade treatment and the survival outcomes of patients may be more closely related to the TCR-HLA interactions. While for the TCGA cohort, the treatments that the patients received can be diverse, which, combined with relatively small sample size per cancer type, may have contributed to limited findings. We have added these results in Supplementary Tables F-I, and added a brief discussion in the Discussion Section.

Table 6: Situations with p-value < 0.05 from running Cox regression on TCGA data, with PFI as survival endpoint.

covariates	cancer type	HLA-I metric	pvalue
age+stage+log1p(mut)	LUAD	mean_DePTH_cor	0.0058
age+stage+log1p(mut)	LUAD	DePTH_breadth	0.0207
age+stage+log1p(mut)	LUSC	mean_AA	0.0133
age+stage+log1p(mut)	LUSC	mean_DePTH_set	0.0214

References

[1] Szeto C, Lobos CA, Nguyen AT, Gras S. TCR recognition of peptide–MHC-I: Rule makers and breakers. International journal of molecular sciences. 2020;22(1):68.

[2] Liu J, Lichtenberg T, Hoadley KA, Poisson LM, Lazar AJ, Cherniack AD, et al. An integrated TCGA pan-cancer clinical data resource to drive high-quality survival outcome analytics. Cell. 2018;173(2):400–416.
